# Next-Generation Immunotherapies to Improve Anticancer Immunity

**DOI:** 10.3389/fphar.2020.566401

**Published:** 2021-01-11

**Authors:** Yaoyao Shi, Katarzyna Tomczak, June Li, Joshua K. Ochieng, Younghee Lee, Cara Haymaker

**Affiliations:** Department of Translational Molecular Pathology, The University of Texas MD Anderson Cancer Center, Houston, TX, United States

**Keywords:** DC, B cells, NK cells, neutrophils, combination immunotherapy

## Abstract

Checkpoint inhibitors are widely used immunotherapies for advanced cancer. Nonetheless, checkpoint inhibitors have a relatively low response rate, work in a limited range of cancers, and have some unignorable side effects. Checkpoint inhibitors aim to reinvigorate exhausted or suppressed T cells in the tumor microenvironment (TME). However, the TME contains various other immune cell subsets that interact to determine the fate of cytotoxic T cells. Activation of cytotoxic T cells is initiated by antigen cross-presentation of dendritic cells. Dendritic cells could also release chemokines and cytokines to recruit and foster T cells. B cells, another type of antigen-presenting cell, also foster T cells and can produce tumor-specific antibodies. Neutrophils, a granulocyte cell subset in the TME, impede the proliferation and activation of T cells. The TME also consists of cytotoxic innate natural killer cells, which kill tumor cells efficiently. Natural killer cells can eradicate major histocompatibility complex I-negative tumor cells, which escape cytotoxic T cell–mediated destruction. A thorough understanding of the immune mechanism of the TME, as reviewed here, will lead to further development of more powerful therapeutic strategies. We have also reviewed the clinical outcomes of patients treated with drugs targeting these immune cells to identify strategies for improvement and possible immunotherapy combinations.

## Introduction

**FIGURE 1:**
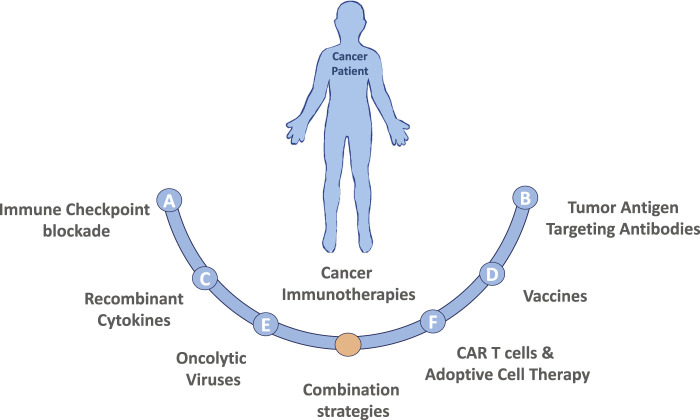
Major types of cancer immunotherapies. **(A)** Immune Checkpoint blockade therapy utilizing antibodies targeting CTLA-4 or PD-1/PD-L1 pathway have demonstrated promising results in a variety of malignancies. Additionally, recent studies have identified many other immune checkpoint markers, such as LAG3, TIM3 or TIGIT that could also be targeted. **(B)** Tumor Antigen Targeting Antibodies are laboratory generated, designed to target specific tumor antigens, usually conjugated with a specific drug. Currently the development of polyspecific antibodies (bi- and tri-specific antibodies) has the advantages by targeting multiple tumor antigens, to more precisely and effectively eradicate cancer cells. **(C)** Recombinant Cytokines (e.g., IL-2, IL-18, IL-6, IFNγ, GM-CSF) can induce, mediate and regulate the immune response by improving antigen priming, facilitating T cell proliferation and survival or enhancing their cytolytic activity. **(D)** Therapeutic Vaccines made of laboratory modified cancer cells, parts of cells, or pure antigens elicit an immune response against tumor-specific or tumor-associated antigens. **(E)** Oncolytic viruses (OVs) in the forms of native or engineered viruses can be used to selectively target and kill cancer cells. Advancements of genetic engineering enable successful editing of viral genome of many species to augment antitumor activity and attenuate pathogenicity, but also to express specific cytokines that favor immune cell recruitment and activation or to produce co-stimulatory molecules on tumor cells to facilitate the generation of T-cell activating signals. **(F)** CAR T cells and Adoptive Cell Therapy (ACT) are personalized cancer strategies relying on the collection of immune cellular components from patient, expansion and/or genetically modification of those cells *in vitro* and injection them back to the patient to achieve a therapeutic response. Combination strategies involving the immunotherapies described above as well as combinations including both standard of care chemotherapy or radiation treatment options are also actively being tested in both preclinical models and in the clinical setting.

Cancer immunotherapy harnesses the patient’s own immune system to fight against cancer, distinguishing immunotherapy from conventional cancer therapies, which directly target the tumor cells. Major types of cancer immunotherapies are described in Figure 1. The clinical practice of immunotherapy in cancer patients was initiated by F. Fehleisen and Wilhelm Busch, two German physicians, who noticed that a malignancy shrank after erysipelas infection (Dobosz and Dzieciatkowski, 2019). The preclinical discovery of immune cell subsets and associated cytokines significantly furthered the clinical practice of cancer immunotherapy (Old et al., 1959; Carswell et al., 1975; Herberman et al., 1975; Quesada et al., 1984). Subsequently, checkpoint inhibitors such as cytotoxic T-lymphocyte antigen 4 (CTLA-4) and programmed cell death-1 (PD-1) and its ligand (PD-L1) were found to play a critical role in immune escape (Leach et al., 1996; Dong et al., 2002). Monoclonal antibodies targeting checkpoint inhibitors were approved by the US Food and Drug Administration for clinical treatment of various malignancies and achieved promising therapeutic outcomes in certain cancer types. A pooled analysis of data from phase II and III trials of ipilimumab (anti-CTLA-4) in patients with unresectable or metastatic melanoma revealed a 22% 3-year long-term survival rate with a median overall survival duration of 11.4 months (Schadendorf et al., 2015). In contrast, the 3-year long-term survival rate of patients who receive routine chemotherapy using dacarbazine is 12.2% (Robert et al., 2011). Nivolumab, a PD-1 inhibitor, achieved an objective response rate of 40.0% in patients with melanoma without the *Braf* mutation, 19% in non-small-cell lung cancer (NSCLC), and 66.3–87% in Hodgkin lymphoma (Ansell et al., 2015; Borghaei et al., 2015; Robert et al., 2015; Younes et al., 2016). Pembrolizumab, another PD-1 inhibitor, achieved a 39% objective response rate in patients with locally advanced or metastatic NSCLC, with a PD-L1 tumor proportion score ≥50% (Mok et al., 2019). Chimeric antigen receptor (CAR)-T cell therapy, which targets the specific tumor antigen, was also approved by the US Food and Drug Administration recently and was reported to lead to a remarkable remission rate in patients with B cell acute lymphocytic leukemia (DiNofia and Maude, 2019).

Both checkpoint inhibitors and CAR-T cell therapy have been shown to lead to beneficial clinical outcomes in selected cancer types mentioned above; however, the response rate to checkpoint inhibitors is relatively low. CAR-T cell therapy has a relatively high response rate in hematologic malignancies, but huge challenges exist when CAR-T cell therapy is utilized to treat solid tumors. Specifically, the tumor-specific antigens that are the potential target for CAR-T cell therapy are lacking; meanwhile, the suppressive immune environment of solid tumors limits CAR-T cells’ infiltration into the solid tumors (Ma et al., 2019). Another issue for CAR-T cell therapy is the severe immune-related adverse events, including cytokine release syndrome, neurologic toxicity, and off-tumor recognition (Bonifant et al., 2016). Checkpoint inhibitors, especially anti-CTLA-4 agents, also have relatively high rates of immune-related adverse events, including colitis and hypophysitis (Postow et al., 2018).

**FIGURE 2:**
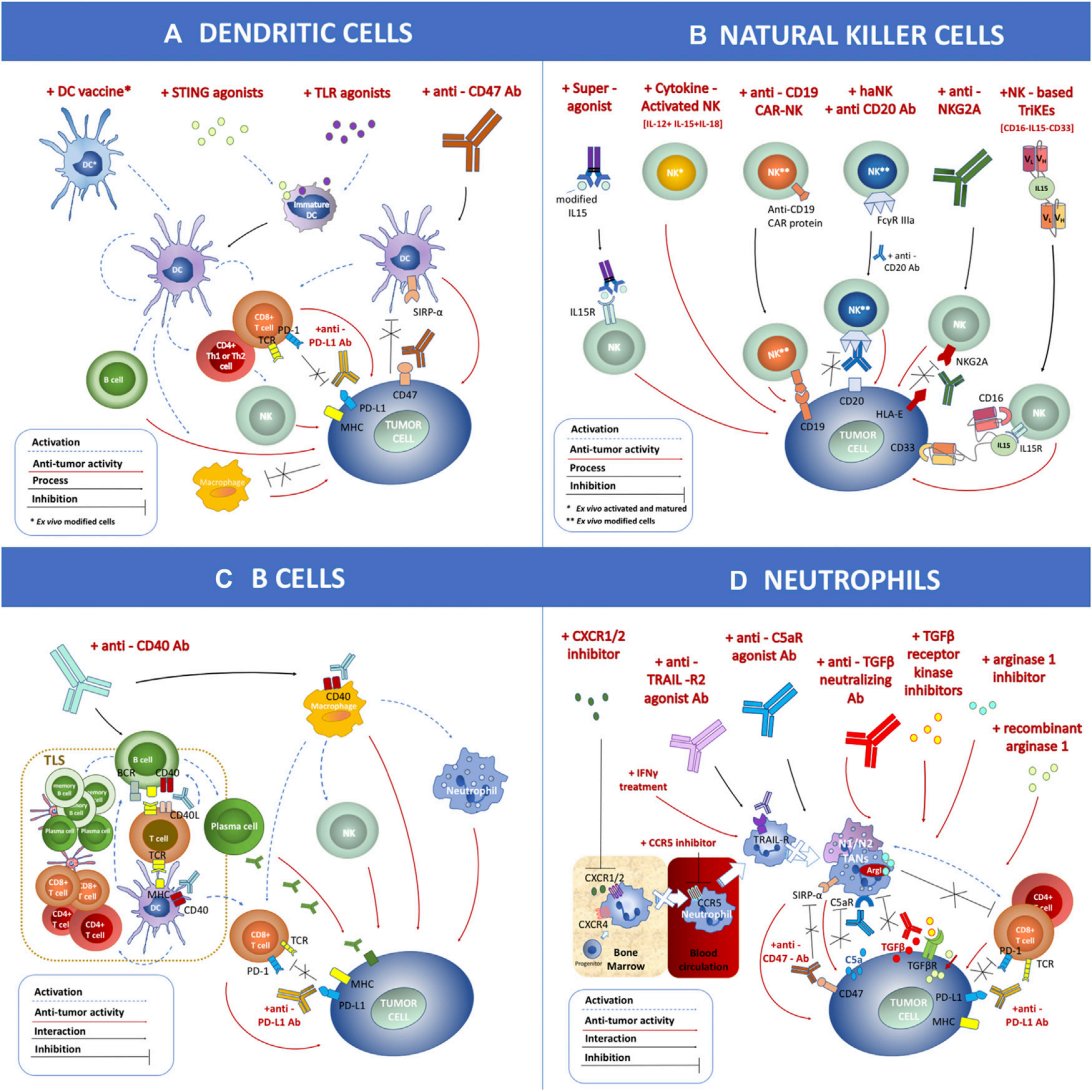
Schematic overview of immune cell-mediated anticancer therapies based on dendritic cells (DCs), natural killer (NK) cells, B cells, and neutrophils applied within clinical trials. **(A)** DC-related antitumor immunotherapies include the DC vaccine loaded with tumor antigen, STING agonist, TLR agonist, and anti-CD47 antibodies. **(B)** NK cell-related antitumor immunotherapies include treatment with a superagonist, cytokine-activated NKs, anti-CD19 chimeric antigen receptor-NK, combination of haNK with anti-CD20 antibody, application of anti-NKG2A antibody, and NK-based CD16-IL15-CD33 TriKEs. **(C)** B cell-related antitumor immunotherapies include treatment with anti-CD40 antibody. **(D)** Neutrophil-related antitumor immunotherapies include CXCR1/2 inhibitor, CCR5 inhibitor, anti-TRAIL-R2 agonist antibody, anti-C5aR agonist antibody, treatment with IFNγ, anti-TGFβ neutralizing antibody, TGFβ receptor kinase inhibitor, arginase 1 inhibitor, recombinant arginase 1, and anti-CD47 antibody. These strategies can be combined with other therapies such as anti-PD-L1 antibody. The antitumor effect (marked as a red dashed arrow) or inhibitory interaction (marked as a black line) is indicated within the figure.

Both checkpoint inhibitors and CAR-T cell therapy aim at invigorating T cells’ response to fight against cancer. However, the tumor immune microenvironment (TME) contains multiple types of immune cell subsets, not limited to T cells. The multilevel and multiscale interactions among these immune cells determine their final capacity of tumor control (Chew et al., 2012). In this review, we describe four of these immune cell types, i.e., dendritic cells (DCs), natural killer (NK) cells, B cells, and neutrophils, along with current methods to target these cells to induce antitumor immune activation (Figure 2). DCs are the “professional” tumor antigen-presenting cells, which essentially initiate the activation of T cell response in the TME (Banchereau and Palucka, 2005). Similar to cytotoxic T cells, NK cells are another powerful cytotoxic immune cell capable of direct tumor killing, and NK cells do not need prior exposure to the specific antigen (Morvan and Lanier, 2016). B cells, another cell subset in the adaptive immune system, are required for optimal T cell activation in the TME or tumor-draining lymph nodes (Dilillo et al., 2010). Most neutrophils in the TME, conversely, suppress T cell function and promote tumor progression and metastasis (Wu et al., 2019). The direct tumor killing capability or the significant impact on cytotoxic T cell function make these immune cells the potential next-generation cancer therapeutic targets. The combination of therapies targeting these cells with checkpoint inhibitors or CAR-T cell therapy may significantly improve clinical outcomes for various cancer types.

## Dendritic Cells

Most current immunotherapies, including checkpoint inhibitors, aim to invigorate the adaptive immune response, specifically cytotoxic T cells, to fight against cancers. However, an antitumor adaptive T cell response is not triggered autonomously. Instead, it is initiated, activated, and regulated predominantly by one innate immune cell subset: DCs (Patente et al., 2018). DCs are professional antigen-presenting cells, capturing tumor-associated antigens (TAA) in the TME. Distinct from macrophages, which destroy TAAs completely into amino acids for the purpose of antigen clearance, DCs partially degrade TAAs into potential peptides for T cell recognition (Ramachandra et al., 2009). Specifically, TAA peptides are presented by the major histocompatibility complex (MHC) I molecule on the surface of DCs to be recognized by TAA-specific T cells. This is known as cross-presentation. After this process, neoantigen-specific CD4^+^ T helper cells and cytolytic CD8^+^ T cells are activated and start to fight against tumor cells (Neefjes et al., 2011). In addition, DCs can produce cytokines such as tumor necrosis factor alpha (TNFα) and type I interferon (IFN) in an autocrine manner to further promote DC activation and maturation (Blanco et al., 2008). DCs can also secrete chemokines such as CCL2/3/4, CCL17, CCL19/21, and CXCL9/10/11 to recruit T cells and other cell subsets into the tumor sites (Thaiss et al., 2011). DCs can also activate B cells, NK cells, and NK T cells (Fernandez et al., 1999; Kadowaki et al., 2001; Jego et al., 2003), thereby conducting all elements of the immune orchestra in concert to eliminate tumors.

However, DC function is suppressed in the TME. During the process of tumor formation, tumor cells manipulate immune cells, including DCs, to form a suppressive immune environment to foster tumor cell expansion. Immunosuppressive regulatory DCs that secrete interleukin (IL)-6 and galectin-1 to promote tumor growth have been observed in the TME (Veglia and Gabrilovich, 2017). In addition, DCs in patients with breast cancer were reported to express lower MHC II and maturation markers and to be less effective in stimulating cytotoxic T cells than in healthy donors (Gervais et al., 2005). Patients with renal cell carcinoma were reported to have minimal recruitment and activation of DCs in the TME (Troy et al., 1998). Reinvigoration of DC function will be a powerful step in restarting the robust antitumor adaptive immune response in patients with cancer (Figure 2A).

DCs have been explored in clinical trials as a target for therapeutic vaccination of patients with cancer since the mid-1990s (Anguille et al., 2014). Specifically, autologous DCs, *ex vivo* loaded with specific TAAs or peptides or pulsed with whole tumor lysate, were used as a therapeutic vaccine (Chiang et al., 2015). The sources of autologous DCs include peripheral blood monocytes, circulating DCs after *in vivo* expansion, or CD34^+^ hematopoietic precursors mobilized from bone marrow (Constantino et al., 2017). DC vaccines have been extensively tested in clinical trials of patients with malignant melanoma, prostate cancer, malignant glioma, and renal cell cancer. In patients with melanoma, DC vaccines resulted in an objective response rate of 8.5%, and median overall survival was prolonged by at least 20% in most trials (Anguille et al., 2014). Similarly, 7.1% of patients with prostate cancer had an objective response. Notably, the phase III clinical trial of DC-based therapeutic sipuleucel-T showed a 4.1-month improvement in median overall survival in patients with prostate cancer (Kantoff et al., 2010). In addition, 15.6% of patients with malignant glioma had an objective response to the DC vaccine and 11.5% of patients with advanced renal cell carcinoma had an objective response (Martin Lluesma et al., 2016). In brief, the overall clinical benefit of the DC vaccine is real but underwhelming. Unlike with other immunotherapies, immune-related adverse events with DC vaccination are not severe, and systemic grade 3–4 toxicity is uncommon with monotherapy (Draube et al., 2011).

Cancer therapies targeting *in vivo* DC maturation and activation have also been investigated and put into clinical practice. Toll-like receptors (TLRs) are one molecule family expressed by DCs. The activation of TLR signaling in DCs enhances antigen cross-priming of naïve T cells and upregulates cytokine production to further promote DC maturation in an autocrine manner (Hemmi and Akira, 2005). TLR7 and TLR9 agonists, which target plasmacytoid DCs to produce type I IFN, showed clinical efficacy. In a multicenter phase III study using imiquimod (a TLR7 agonist) to treat basal cell carcinoma, patients using imiquimod had 80% histologic tumor clearance compared with 6% clearance in control groups (Schulze et al., 2005). In an early-phase trial of IMO-2055 (a TLR9 agonist) to treat NSCLC, 15% of patients achieved a partial response and 61% had stable disease (Smith et al., 2014). Like TLRs, the stimulator of IFN genes (STING) signaling in DCs can also induce type I IFN expression and promote intrinsic or therapy-induced antitumor T cell responses (Vatner and Janssen, 2019). The first notable clinical STING agonist, vadimezan, was shown to stimulate mouse STING and boost antitumor immunity in preclinical studies; however, vadimezan cannot bind human STING and thereby failed to improve antitumor efficacy in a randomized phase III trial conducted in patients with advanced NSCLC (Lara et al., 2011; Conlon et al., 2013). Afterward, various human STING agonists such as ADU-S100 and MK-1454, which may have promising clinical outcomes, were developed and are currently being tested in combination with checkpoint inhibitors in ongoing clinical trials (NCT02675439, NCT03172936, NCT03010176).

Another suppressive factor in the TME that results in DC anergy is the “do not eat me” molecules expressed on tumor cells. DCs in the TME failed to recognize tumor cells expressing CD47, one of the “do not eat me” molecules (Liu et al., 2015). Magrolimab (a CD47-blocking antibody) is being tested in various ongoing clinical trials (NCT02953509, NCT04313881, NCT03248479), and promising preliminary data from NCT02953509 showed that 40% of patients with non-Hodgkin lymphoma achieved an objective response and 33% achieved a complete response when magrolimab was combined with rituximab (Advani et al., 2018).

In the TME, DCs not only continuously present TAAs to initiate robust T cell responses but also coordinate with other immune cell subsets, including NK cells and B cells, to eliminate cancer cells. The current pitfall for DC vaccination lies in its limited efficacy in terms of tumor control (Anguille et al., 2014). The antitumor efficacy of the DC vaccine has been reported to partially depend on the maturation status of the DCs. Results from prostate cancer clinical trials showed that DCs derived from mature monocytes led to better clinical outcomes than did their immature counterparts (Draube et al., 2011). The current gold standard method to induce *in vitro* DC maturation is using a pro-inflammatory cytokine mix (IL-1, TNFα, IL-6, and prostaglandin E). More efficient methods to induce DC maturation have also been developed, such as adding type I/II IFN, CD40, or TLR agonist (Banchereau and Palucka, 2005). Homing efficiency of vaccinated DCs in the tumor-draining lymph node(s) is also critical to optimize the clinical outcome of patients who receive the DC vaccine (Shang et al., 2017). Various administration methods have been tested to achieve higher levels of infused DC infiltration in the tumor-draining lymph nodes and TME (Perez and De Palma, 2019). Antigen loading efficiency presents another problem for DC vaccination. DCs pulsed by TAA peptides are the most common DC vaccines in clinical practice. However, the low conjugation affinity of these peptides with MHC molecules and the fast turnover of the peptide-MHC complex on DCs reduces the antitumor efficacy of DC vaccines (Banchereau and Palucka, 2005). Therefore, methods to achieve long-lasting and natural processing of the peptide-MHC complex is an area for further exploration and improvement.

As for immunotherapies to activate DCs *in vivo*, TLR agonists, STING agonists, or anti-CD47 antibodies can activate DC function and turn “cold tumors” into “hot tumors.” However, the administration of these stimulators leads to upregulation of type I and II IFN in the TME, resulting in upregulation of PD-L1 expression, causing T cell suppression/exhaustion (Blanco et al., 2008; Liu et al., 2015; Garcia-Diaz et al., 2017; Vatner and Janssen, 2019). Therefore, the logical combination of these DC stimulators with checkpoint inhibitors may lead to a more favorable clinical outcome in patients with cancer.

## NK Cells

NK cells are a powerful subset of innate cytotoxic lymphocytes fighting against pathogens and tumor cells, without the need for prior exposure to a specific antigen (Morvan and Lanier, 2016). NK cells can lyse cancer cells through secretion of cytolytic granules, such as perforin and granzyme, and foster other immune responses via secretion of immunomodulatory cytokines. NK cells can also induce tumor cell apoptosis by releasing members of the TNF family, such as FAS and TRAIL (Lanier, 2008; Guillerey et al., 2016). Crosstalk between NK cells and DCs promotes DC uptake of tumor antigens in tumor-draining lymph nodes, thereby activating T cells. Recently, it was also found that, like adaptive immune cells, NK cells can acquire features of memory-like responses, under certain circumstances (O'Sullivan et al., 2015; Rapp et al., 2018). The activity of NK cells depends upon a delicate integration of signals from multiple surface-activating and inhibitory receptors on NK cells that cognate with ligands on target cells. Inhibitory receptors, such as killer cell immunoglobulin-like receptors (KIRs) and CD94-NK group 2A (NKG2A), could be conjugated by MHC class I to inhibit NK cell activity. The MHC class I molecule is constitutively expressed on normal cells but is often downregulated or lost in malignant cells. Therefore, tumor cells can be distinguished from normal cells by NK cells, through the process of “missing-self recognition” (Raulet, 2006). Activating receptors on NK cells, such as NKG2D, NKG2C, and natural cytotoxicity receptors NKp30, NKp44, and NKp46, bind to their corresponding ligands, resulting in NK cell activation.

NK cell activity is frequently suppressed in both hematopoietic malignancies and solid tumors owing to downregulation of activating receptors, such as NKp30 and DNAM-1, and upregulation of inhibitory receptor(s), such as KIR and CD94/NKG2A. In addition, leukemic blasts express more ligands to the inhibitory receptors mentioned above. In solid tumors, NK cells also highly express immune checkpoint receptors shared with T cells, including CTLA-4, T cell immunoglobulin- and mucin-domain-containing molecule 3 (TIM-3), lymphocyte activation gene 3 (LAG-3), T cell immunoreceptor with immunoglobulin and immunoreceptor tyrosine-based inhibition motif domains (TIGIT), and CD96 (Del Zotto et al., 2017; Pesce et al., 2019; Sivori et al., 2019; Sun and Sun, 2019). Moreover, the presence of NK cells in the tumor tissue is rare. These features contribute to varying degrees to the suppression of NK cell activity. NK cells’ natural toxicity and broad target cell reactivity make NK-cell-based therapy an alternative or complementary immunotherapy approach to T cell therapy. Given the cell biology of NK cells, various approaches designed to bolster NK cell activity against cancer have been tested in clinical trials and are described below (Figure 2B).

### Clinical Trials of Cytokine-Activated NK Cells

Pre-activation of human peripheral blood-derived NK cells using a cytokine cocktail mix, such as IL-18, IL-15, and IL-13, can induce human NK cell differentiation into memory-like NK cells with features of increased proliferative capacity, long-term survival and *in vivo* persistence, enhanced cytokine production, and increased cytotoxicity (Romee et al., 2012; Pahl et al., 2018). Recently, several phase I and II clinical trials of cytokine-induced memory-like NK cells have been performed in patients with relapsed or refractory acute myeloid leukemia (AML) after allogeneic hematopoietic cell transplantation. Favorable clinical responses have been observed, including improved survival in the absence of graft-versus-host disease (GVHD), which is associated with donor NK cell expansion and the graft-versus-leukemia effect. More recently, a phase I/II clinical trial of haploidentical NK cell infusion given with recombinant human IL-15 resulted in remission in 35% of patients with refractory AML, with *in vivo* NK cell expansion (Cooley et al., 2019), indicating that persistence and expansion of these NK cells *in vivo* is key for achieving a clinical response and that IL-15 alone is powerful enough to make this happen. However, although large-scale expansion of these NK cells is possible, and the graft-versus-leukemia effect against leukemic cells or tumor cells without accompanying GVHD is a big advantage of these NK cells, they are undesirable for the treatment of solid tumors.

Patients’ endogenous NK cells can be activated by administration of IL-15. IL15-IL15Rα-Sushi-Fc fusion proteing (ALT-803), termed superagonist, in which the IL-15Ra sushi domain is complexed with IL-15, is more powerful than native IL-15 for enhancing NK cell activity (Mathios et al., 2016). Phase I clinical studies in patients with various leukemias or solid tumors verified the safety of ALT-803 (Margolin et al., 2018; Romee et al., 2018; Wrangle et al., 2018). A study in patients with AML who experienced relapse after allogeneic hematopoietic cell transplantation demonstrated that ALT-803 significantly increased NK and CD8^+^ T cell numbers and function, with a response rate of 19%, including one complete remission lasting 7 months (Romee et al., 2018).

### Clinical Trials of CAR NK Cells

The CAR offers specificity to NK cells for intended target cells. CAR-NK cells are potentially safer than CAR-T cells because allogeneic infusion of CAR-NK cells has low likelihood of triggering GVHD. CAR-NK cells seldom result in cytokine release syndrome because NK cells mainly secrete IL-3, IFNγ, and granulocyte-macrophage colony stimulating factor (CSF), not pro-inflammatory cytokines IL-1, IL-6, and TNFα (Liu et al., 2020; Wang et al., 2020). However, the ability of CAR-NK cells to penetrate tumor tissue is inferior to that of CAR-T cells. In addition, genetic manipulation of primary NK cells is more challenging. The targets used for CAR-NK research in preclinical studies include CD19, CD20, CD138, CD5, CD2 subset 1 (CS1), NKG2D ligand, glucosylceramidase beta (GD2), HER2, epidermal growth factor receptor (EGFR), EGFRvIII, epithelial cell adhesion molecule 1, glypican 3, and guanine nucleotide-binding protein alpha-7 to target distinct cancer cell types (Rezvani et al., 2017). CD19 CAR-NK cells have shown remarkable clinical efficacy in B cell cancers. In a phase I/II trial, human leukocyte antigen-mismatched anti-CD19 CAR-NK cells derived from cord blood were infused into 11 patients with relapsed or refractory CD19-positive cancers; 73% of patients (8/11) had a response and seven patients had a complete remission without development of major toxic effects (Liu et al., 2020). Several CAR-NK cells (targeting CD19, CD22, CD7, and CD33) are currently in clinical trials for the treatment of certain types of leukemia or lymphoma (e.g., NCT03056339, NCT04004637).

### Clinical Trials of NK Cells With Enhanced Antibody-Dependent Cellular Cytotoxicity

When anticancer antibodies target cancer cells by binding to a cancer antigen, the Fc region of the antibodies may recruit NK cells by binding to CD16 (FcγRIII) on NK cells, triggering Antibody-Dependent Cellular Cytotoxicity (ADCC), by which tumor-targeting antibody drugs exert their antitumor effects (Pereira et al., 2018). Because interaction between FcγRIII and Fc occurs naturally and is not necessarily tight, in recent years, several approaches have been used to increase the affinity between FcγRIII and Fc by modifying antibodies or FcγRIII, enhancing ADCC-mediated NK cell antitumor effects. haNK, a NK-92 cell line engineered with high-affinity FcγRIIIa (158V) allele (Gleason et al., 2012), has been tested in phase I and II clinical trials, either alone or in combination with anti-PD-L1 antibody (avelumab), a cancer vaccine, or super-IL-15. Most of these trials are ongoing for the treatment of triple-negative breast cancer, squamous cell carcinoma, Merkel cell carcinoma, pancreatic cancer, and other types of cancers. Phase I trials of CAR-modified haNK (also named target-activated NK-92) cells targeting CD19 in patients with B cell lymphoma or PD-L1 in patients with solid tumors are currently ongoing (NCT04052061, NCT04050709). Margetuximab, against HER2, with Fc-engineered and subsequently elevated affinity to CD16A, was shown to be well tolerated and highly effective in patients with HER2-overexpressing carcinomas (Bang et al., 2017). Of the 60 patients in this trial, partial response was observed in seven patients (12%) and stable disease in 30 (50%); 78% of patients (18/23) had tumor reductions. Both obinutuzumab (GA101) and ublituximab are afucosylated antibodies against CD20, in which Fc is modulated, leading to enhanced binding affinity for FcγRIIIa and increased ADCC activity. Phase I/II trials demonstrated that both drugs are safe and efficacious in patients with B cell malignancies. In phase III trials, GA101 in combination with chlorambucil prolonged overall survival significantly, as well as prolonging progression-free survival and increasing the complete response rate (Pereira et al., 2018).

### Clinical Trials of Enhanced NK Cell Activity by Targeting Inhibition Receptors and Immune Checkpoint Molecules

The TME is a major obstacle for optimizing the antitumor activity of NK cells, because in the TME immunosuppressive cells and molecules limit NK cell function through downregulation of activating receptors, upregulation of inhibitory receptor(s), and upregulation of immune checkpoint receptors on NK cells. In an effort to restore NK cell activity against tumor cells, researchers have developed anti-NKG2A (monalizumab/IPH2201) and anti-pan-KIR2D (lirilumab/IPH2102) antibodies for blockage of these inhibitory receptors. Phase I/II clinical trials showed that monalizumab/IPH2201 alone was well tolerated but resulted in short-term disease stabilization as the best response in patients with various advanced gynecologic malignancies. Combination therapy comprising IPH2201 and cetuximab (anti-EGFR antibody) enhanced antitumor immunity (Tognarelli et al., 2018). An overall response rate of 31% and a disease stabilization rate of 54% were obtained, in patients with squamous cell carcinoma of the head and neck (Tinker et al., 2019). The anti-pan-KIR2D agent lirilumab/IPH2102 was examined in several phase I/II clinical trials. It was well tolerated in patients with hematologic malignancies or solid tumors. Combination therapy with the anti-PD-1 antibody nivolumab or with the anti-CTLA-4 antibody ipilimumab in a population of patients with various solid tumors showed a durable response, and an overall response rate of 24% was achieved in 29 patients with squamous cell carcinoma of the head and neck (Vey et al., 2018). Other phase I clinical trials of combination therapies for various solid tumors are ongoing (NCT03532451, NCT03341936).

Because NK cells express some immune checkpoint molecules shared with T cells, including PD-1, CTLA-4, TIM3, LAG3, and TIGIT, checkpoint inhibitors targeting these molecules should enhance the function of both T cells and NK cells against cancer. Currently, multiple phase I/II clinical trials in patients with solid tumors or hematologic malignancies are ongoing for these checkpoint inhibitors, including anti-TIM3 (e.g., NCT03489343), anti-LAG3 (e.g., NCT03005782), and anti-TIGIT (e.g., NCT04354246), as well as combination therapies, such as the combination of anti-TIM3, anti-PD-1, and anti-LAG3 (NCT04370704) and the combination of anti-LAG3 with anti-TIGIT (NCT04150965).

### Clinical Trials of Bi- or Tri-Specific Killer Engagers for NK Cells

Bi-Specific Killer Engagers (BiKEs) and Tri-Specific Killer Engagers (TriKEs) are multi-specific antibodies composed of two to three single-chain variable fragments of antibodies with different specificities, joined together by a short peptide linker. Usually, one of the fragments is directed against CD16 on NK cells to induce NK cell–mediated ADCC. BiKEs and TriKEs can boost NK cell activity and promote NK-mediated killing of tumor cells because they create an immune connection between NK and tumor cells (Gleason et al., 2012; Felices et al., 2016).

Several NK-based BiKEs or TriKEs are currently in preclinical and clinical development. CD16-directed BiKEs CD16 × CD19 and CD16 × CD33 and TriKE CD16 × CD22 × CD19 were shown to specifically stimulate NK cell activation via CD16, which triggers NK cell cytolytic activity and secretion of cytokines to attack CD19^+^, CD33^+^, and CD19^+^CD22^+^ lymphoma and leukemia, respectively (Gleason et al., 2012; Nagato et al., 2017). CD16 × IL-15 × CD33 TriKE displayed markedly enhanced NK cytotoxicity against AML and better NK cell persistence than did BiKE CD16 × CD33 *in vivo*, because the TriKE provided NK cell expansion signal via IL-15 moiety. CD16 × IL-15 × CD33 TriKE is being evaluated in phase I and II clinical trials in patients with advanced systemic mastocytosis, relapsed or refractory AML, or CD33-expressing high-risk myelodysplastic syndromes NCT03214666 (Wiernik et al., 2013; Schmohl et al., 2016; Schmohl et al., 2017). TriKEs targeting two activating receptors, NKp46 and CD16, on NK cells and a tumor antigen (CD19, CD20, or EGFR) on cancer cells have been generated in the laboratory (Gauthier et al., 2019). TriKEs represent a versatile platform for incorporation of various targeting molecules and will be a promising tool for NK cell immunotherapy.

NK cell–based anticancer therapy has achieved clinical benefits for various cancer types, especially hematologic malignancies. CAR-NK cell therapy still faces critical challenges, including difficulty in genetic manipulation of primary NK cells and difficulty in storage of CAR-NK cells (Wang et al., 2019b). Currently, more cell resources, such as human NK cell lines, human embryonic stem cells, induced pluripotent stem cells, and bone marrow or umbilical-cord blood, are being tested as alternative sources of therapeutic NK cells (Wang et al., 2019a). There are also some challenges for solid tumors because NK cells have difficulties in trafficking and infiltrating into solid tumor sites. Therefore, future directions for NK cell development will include enhancing activating signals, suppressing inhibitory signals, and promoting NK cell homing to tumors.

## B Cells

Bursal-derived lymphocytes (B cells) arise from hematopoietic stem cells residing within the spongy bone marrow and have a significant impact on the TME through their antibody production and antigen presentation capabilities (Shahaf et al., 2016). Currently, the potential of B cells for cellular therapy is still largely underestimated despite their multiple diverse effector functions.

The antitumor response of B cells has been linked to tumor-infiltrating B cells and the formation of tertiary lymphoid structures (TLSs), which correlate with favorable clinical outcome in patients treated with immune checkpoint blockade (LeBien and Tedder, 2008; Adamo et al., 2020). TLSs are composed of T cells and mature DCs located in the T cell–rich areas close to a B cell follicle, a setting that suggests a local antigen-driven antibody response resulting in production of antibodies with antitumor or pro-tumor properties (Romero, 2020). Thus, TLSs represent a potential for T cell priming, B cell activation, and B cell differentiation into plasma cells and an intricately located factory for antibody production (Teillaud and Dieu-Nosjean, 2017). Considering the multiple roles of tumor-infiltrating B cells in tumor immunity, B cell depletion therapy, and selective clearance of regulatory B cells, promoting TLS formation and targeted regulation of tumor-infiltrating B cell-linked signaling pathways may become effective strategies for tumor-infiltrating B-cell-based tumor immunotherapy.

### B Cell Antigen Presentation

B cells recruit and activate T cells in a cognate or non-cognate manner and trigger T cell polarization, impacting T cell-mediated antitumor responses (Teillaud and Dieu-Nosjean, 2017). TLS-B cells present features of B cell follicles marked by homing of naïve and germinal center B cells, with scattered plasmablasts and memory B cells (Teillaud and Dieu-Nosjean, 2017; Helmink et al., 2020). TLS-associated antibody responses are speculated to be elicited by TAAs, suggesting that TLSs are critical for the development of efficient B cell–dependent antitumor immunity (Germain et al., 2014).

The immunostimulatory capacity of B cells requires B cell receptor binding to antigen and TLR-mediated signals (Wennhold et al., 2019). The CD40L/CD40 signaling pathway is a potent activator of antigen presentation capacity in B cells (Van Belle et al., 2016). B cell activation by CD40-mediated signals has been shown to be affected by the location of B cell binding to CD40 and the degree of CD40 crosslinking (Barr and Heath, 2001). Additionally, stimulation of CD40 results in improved antigen processing and presentation via the MHC class II pathway (Faassen et al., 1995). CD40 B cells equally cross-present antigens via MHC class I pathways and, thus, induce naïve and memory CD8^+^ T cell responses (Wennhold et al., 2019). Tumor-infiltrating B cells have been identified in association with TLSs; however, their functions remain elusive. TLSs are thought to modulate antitumor immune activity; mature TLSs exhibit evidence of formation of germinal centers (Selitsky et al., 2019). Some strategies for modulating B cell activity within the TME are described in the sections below and in Figure 2C.

### B Cell-Based Therapies

A wide range of preclinical studies using immunocompetent cancer mouse models so far underscore CD40-directed therapies as a next-generation immune-modulating therapy (Piechutta and Berghoff, 2019). Therapeutic strategies associated with B cells focus on CD40 because the ligation of CD40 with CD40L on helper T cells is critical for antigen-presenting cell activation and proliferation and for immunoglobulin class-switch (Eltahir et al., 2020). Preclinical data have demonstrated that CD40-activated B cell-based cancer immunotherapy induces effective antitumor immunity; however, CD40 agonistic antibodies rely on combination therapy strategies (Wennhold et al., 2019). Moreover, studies have shown that agonistic CD40 therapy can be combined with anti-PD-1 to trigger effective T cell immunity (Gravbrot et al., 2019). Patients with metastatic pancreatic cancer treated with PD-1/PD-L1 checkpoint inhibitors in combination with CTLA-4 and anti-CD40 have shown better responses than those who received PD-1/PD-L1 checkpoint inhibitors alone. Additional studies have also demonstrated that patients with pancreatic ductal adenocarcinoma had their tumors dramatically shrink when treated with CD40 monoclonal antibodies in combination with chemotherapy (Helmink et al., 2020).

Ongoing clinical studies investigating agonistic CD40 antibodies focus on the activity of these antibodies in enhancing the CD40/CD40L axis. The initial clinical study CP-870,893 (selicrelumab, Hoffman-La Roche) reported a maximum tolerated dose of 0.2 mg/kg body weight as a single infusion and showed that this dose led to a partial response rate of 13.8% (4 of 29 patients) in a phase I study (Vonderheide et al., 2007). A follow-up study applied selicrelumab on a weekly basis and provided further evidence of safety for the previously determined maximum tolerated dose. However, that study failed to reproduce the promising clinical data of the first study (7 of the 27 enrolled patients (Ruter et al., 2010). The combination of selicrelumab with gemcitabine/nab-paclitaxel as neoadjuvant and adjuvant therapy for pancreatic adenocarcinoma (NCT02588443) is currently under investigation in an ongoing phase I study.

Additionally, the therapeutic potential of selicrelumab is being investigated in three additional clinical phase I trials investigating the efficacy of the drug in patients with advanced solid tumors. In these studies, selicrelumab is combined with another immune-modulating or targeted antibody, including i) emactuzumab, an anti-CSF1 receptor antibody (NCT02588443); ii) vanucizumab, a bispecific antibody targeting angiopoietin 2 and vascular endothelial growth factor (VEGF), or bevacizumab, an anti-VEGF monoclonal antibody (NCT02665416); and iii) atezolizumab, an anti-PD-L1 monoclonal antibody (NCT02304393 (Piechutta and Berghoff, 2019).

Other clinical trials include APX005M, a second-generation anti-CD40 agonistic antibody with improved FcγRIIb. Compared with the first-generation antibodies, it contains a non-fucosylated Fc region with cross-linking capacity that improves tumor immunity (White et al., 2015). APX005M has so far been investigated in nine early clinical trials. The dose-finding phase I study completed recruitment at the end of 2018 (NCT02482168). Three phase II studies aiming to test the safety and clinical efficacy of APX005M in combination with standard of care in several solid cancer types (metastasized pancreatic adenocarcinoma: NCT03214250; advanced soft tissue sarcoma: NCT03719430; resectable gastroesophageal carcinoma: NCT03165994) are currently recruiting. In addition, investigation of the safety and antitumor efficacy of APX005M is ongoing in melanoma patients and those with NSCLC, with the treatment applied either systemically (NCT03123783) or intratumorally (NCT02706353), in combination with PD-1 blockade.

The combination of APX005M with cabiralizumab, an anti-CSF1 receptor antagonist, with and without nivolumab (NCT03502330) in patients with melanoma, NSCLC, or renal cell carcinoma whose treatment with anti-PD-1 or anti-PD-L1 had failed (i.e., in order to overcome anti-PD-1/anti-PD-L1 therapy resistance) is being investigated in a phase I study. Other innovative studies utilizing APX005 to boost the effectiveness of a personalized vaccine (NEO-PV-01) approach with or without checkpoint blockade in patients with advanced melanoma (NCT03597282) are also ongoing. A phase I study of APX005M for recurrent or refractory pediatric brain tumors and newly diagnosed diffuse intrinsic pontine glioma for patients younger than 21 years was launched, representing the first and only study so far to target the CD40/CD40L axis in childhood cancer (NCT03389802).

Other second-generation CD40 agonistic antibodies presently under investigation in phase I trials include JNJ-64457107 (NCT02379741, NCT02829099) and SEA-CD40 (NCT02376699). Furthermore, a bispecific antibody, ABBV-428, targeting CD40 and the well-known tumor antigen mesothelin, is currently under investigation in a phase I trial (NCT02955251).

To this end, localization of B cells within TLSs has been shown to be enriched in the tumors of responders, thus providing insights into the potential role of B cells and TLSs in the response to immune checkpoint blockade (Cabrita et al., 2020; Helmink et al., 2020; Petitprez et al., 2020). Therefore, linking B cell activation and the presence of TLSs to function in tumors would inform strategies to design new therapies.

## Neutrophils

Neutrophils lead the body’s front line in fighting against pathogens, such as fungi or bacteria. They act like surveillance cells that sweep through the bloodstream to screen the tissue for potential infections or other inflammatory events, such as cancer (Segal, 2005; Mantovani et al., 2008). Because neutrophils originate from bone marrow myeloid precursors, their release into the blood and finally to the site of inflammation or tumor must occur via a stepwise process coordinated by the release of cytokines and chemokines (Furze and Rankin, 2008). Multiple and heterogeneous neutrophil subsets have been identified both in circulation and tissue (Fridlender et al., 2009; Sagiv et al., 2015). The neutrophils infiltrating into tumor sites, so-called tumor-associated neutrophils (TANs), can be identified as (CD62L^lo^CD54^hi^). TANs are further characterized by a repertoire of chemokine receptors, such as CCR5, CCR7, CXCR3, and CXCR4. Furthermore, when compared with blood-circulating neutrophils, TANs not only exhibit distinct receptor expression signatures but also produce substantial quantities of the proinflammatory factors MCP-1, IL-8, MIP-1α, and IL-6 and anti-inflammatory IL-1R antagonist (Eruslanov et al., 2014; Gabrilovich, 2017). Of note, TANs can display diverse responses to the tumor depending on the presence of different stimuli, such as type I IFN with TANs polarizing toward antitumor (N1) subset or transforming growth factor beta (TGFβ) toward pro-tumorigenic (N2) subset; (Fridlender et al., 2009; Andzinski et al., 2016; Shaul et al., 2016; Hong, 2017). Further studies confirmed these differential profiles (Shaul et al., 2016). Whereas the N1 subset expresses high levels of TNFα, CCL3, and ICAM-1 and low levels of arginase, the N2 subset is characterized by the upregulation of chemokines CCL2, CCL3, CCL4, CCL8, CCL12, CCL17, CXCL1, CXCL2, IL-8/CXCL8, and CXCL16 (Fridlender and Albelda, 2012). Those notable differences of TAN subsets and their plasticity are important factors to be considered while designing anticancer therapies.

The antitumor activity of the TAN N1 subset is executed via direct or indirect cytotoxicity, as well as through activation of different innate and adaptive immune cells, including T and B lymphocytes, NK cells, and DCs (Gerrard et al., 1981; Mantovani et al., 2008). For example, the cytotoxic effect of the N1 subset is achieved through enhanced NADPH oxidase activity, which leads to the production of reactive oxygen species, causing direct tumor cell apoptosis, granule release, tumor cell sloughing, ADCC through Fc receptors, and trogoptosis (Otten et al., 2005; Hodi et al., 2010; Lohse et al., 2012; Matlung et al., 2018). Studies of the antitumor roles of TANs in mice and humans showed that TANs are involved in the recruitment and activation of intratumor CD4^+^ and cytotoxic CD8^+^ T cells. However, it was also shown that the pro-tumor TAN N2 subset exhibits the ability to suppress the proliferation of intratumor CD8^+^ T cells and their IFNγ production (Coffelt and De Visser, 2016) and to induce CD8^+^ T cell apoptosis by secretion of TNFα and NO (Michaeli et al., 2017). Importantly, blocking the Fas-ligand was shown to augment the effectiveness of checkpoint blockade in *in vivo* cancer models (Zhu et al., 2017). Moreover, the significant role of N2 TANs in tumorigenesis, tumor growth, and metastasis through many neutrophil-derived factors has also been shown (Coussens et al., 2000; Schruefer et al., 2005; Wculek and Malanchi, 2015; Aldabbous et al., 2016; Park et al., 2016; Faget et al., 2017; Loffredo et al., 2017).

The heterogeneity of neutrophil phenotypes and their function and interaction within the TME is complex and not fully understood. Nevertheless, there are many different strategic points that could be harnessed to fight cancer via neutrophil-based therapies (Lecot et al., 2019). Such strategies could target neutrophils at different stages of development, such as maturation, activation, release to the blood stream, migration to the tumor site, and function (Figure 2D). Prospective preclinical studies demonstrated many ways of targeting cancer-related neutrophils (both circulating and tumor-associated), and these studies have paved the way for launching related clinical trials.

### Therapeutic Strategy to Limit the Recruitment of Neutrophils to Tumor Sites

Neutrophils exiting from the bone marrow to the bloodstream could be controlled via inhibition of CXCR2, an important marker for neutrophil migration from the bone marrow into sites of inflammation, or of CXCL4, which acts antagonistically to CXCR2 in allowing neutrophils to exit from the bone marrow (Eash et al., 2010). Genetic or pharmacologic inhibition of CXCR2 in *in vivo* lung and pancreatic cancer models has been shown to significantly decrease Ly6G+ neutrophils owing to their inability to “home,” along with decreasing primary tumor growth and suppressing cancer metastasis (Gong et al., 2013; Steele et al., 2016; Sano et al., 2019). Although loss of CXCR4 results in neutrophil egress from the bone marrow, CXCR4 acquisition is relevant for neutrophil infiltration into the tumor; therefore, inhibiting the CXCL12/CXCR4 axis could also result in therapeutic effects (Xue et al., 2017). The application of reparixin, an inhibitor of CXCR1 and CXCR2, was further evaluated in phase I and II clinical trials. In the phase I clinical trial (NCT02001974), orally administered reparixin with weekly addition of paclitaxel in patients with HER2-negative metastatic breast cancer was demonstrated to be safe, and a 30% response rate was observed (Schott et al., 2017). The phase II study (NCT02370238) is ongoing. Treatment with the CCR5 inhibitor maraviroc, currently used to treat HIV infections, is also being investigated in clinical trials (NCT01736813, NCT03274804) for the treatment of metastatic colorectal cancer. This investigation of maraviroc is based on its previously reported antitumor effects through blocking the release of immature neutrophils from bone marrow and then blocking the recruitment of these immature cells to tumor sites (Velasco-Velazquez et al., 2012; Hawila et al., 2017). Another therapeutic strategy aims at targeting the CD47-SIRPα signaling axis, through either anti-CD47 or anti-SIRPα antibody approaches. This signaling axis is under investigation in multiple clinical trials owing to evidence that blocking this pathway limits neutrophil migration into tumor sites and triggers macrophage-mediated phagocytosis of tumor cells (NCT02216409, NCT03717103, NCT02367196 (Liu et al., 2002; Barrera et al., 2017; Voets et al., 2019).

### Therapeutic Strategies to Deplete Neutrophils

Preclinical studies have demonstrated that neutrophil depletion using an anti-Ly6G approach with progressive selectivity properties may have a therapeutic effect (Daley et al., 2008; Bruhn et al., 2016). However, approaches using Ly6C and Ly6G have also been shown to deplete other immune cells such as monocytes and subsets of CD8^+^ T cells, limiting the selectivity of the treatment and delaying potential pathogen clearance (Bao and Cao, 2011). Ongoing preclinical efforts aim to evaluate the potential synergistic effect of neutrophil depletion with currently approved immunotherapies. Prospective studies using an agonistic TNF-related apoptosis-inducing ligand receptor 2 (TRAIL-R2) antibody have also shown promising results, correlating with increased cell death of mouse granulocytic myeloid-derived suppressor cells *in vitro* and increased effects of CTLA-4 immune checkpoint blockade *in vivo*. A phase I clinical trial (NCT02076451) evaluating the impact of targeting TRAIL-R2 to selectively eliminate myeloid-derived suppressor cells in advanced solid tumors or lymphoma is ongoing (Dominguez et al., 2017). This TRAIL-R2 agonist antibody (DS-8273a) showed selective properties in reducing the circulating fraction of low-density neutrophils without affecting other peripheral blood myeloid and lymphoid cells, and without dose-limiting toxicities. Interestingly, this selective depletion was inversely correlated with the length of progression-free survival. However, the observed decrease was short-term and could not be maintained beyond 28 days from the treatment start. Therefore, further studies including a larger cohort of patients and an extended treatment timeline would be necessary to confirm this treatment outcome. However, although new strategies focus on depletion of neutrophils to decrease tumor growth and improve overall survival, standard strategies using chemotherapy have a common side effect of neutropenia, as characterized by a critical drop in neutrophil blood concentration (Buckley et al., 2014). The ideal targeting of neutrophils in oncology would be to favor the enrichment of antitumor neutrophils while eliminating their pro-tumor counterparts without altering antibacterial neutrophils. One potential way to compensate for the treatment-induced neutropenia is to introduce *ex vivo* manufactured neutrophils (Torres-Acosta et al., 2019).

### Therapeutic Strategies to Target the Suppressive Functions of Neutrophils

Arginase 1 produced by neutrophils was found to suppress T cell proliferation, and depletion of arginase 1 through treatment with an arginase one inhibitor reversed the suppression in a preclinical mouse model (Rodriguez et al., 2004; Marini et al., 2017). However, bioengineered arginase 1 can also exhibit antitumorigenic functions by inducing cell cycle arrest and apoptosis, as was shown in human tumor cell lines and *in vivo* models (Lam et al., 2009; Li et al., 2013). Moreover, the bioengineered human PEGylated arginase 1 (AEB1102) showed additive antitumor effects when combined with anti-PD-1 or anti-PD-L1 in a preclinical *in vivo* model (Agnello et al., 2017; Agnello et al., 2020). The combination of either recombinant arginase 1 or arginase 1 inhibitor with various chemotherapies or immunotherapy (anti-PD-1) is currently being tested and has shown synergistic therapeutic efficacy. Phase I/II clinical trials (NCT03371979; NCT02903914; NCT03361228; NCT03314935) to test the efficacy of this combination are ongoing.

Another targetable feature triggering immunosuppressive activity is C5a receptor (C5aR, CD88). Increased expression of this receptor was found on myeloid-derived suppressor cells and neutrophils, caused by C5a released by cancer cells (Corrales et al., 2012). Pharmacologic inhibition of C5a or blockage of its interaction with the receptor by using antibodies against C5aR showed promising results in many preclinical studies (Ajona et al., 2017; Medler et al., 2018). The blocking antibody against C5aR (IPH5401) in combination with PD-L1 blockade has been tested in clinical phase I trials for the treatment of selected advanced solid tumors (NCT03665129).

### Therapeutic Strategies Modulating the Neutrophil Phenotype

The presence of key players modulating the TAN phenotype, such as TGFβ and IFNβ, promote a phenotype switch toward a pro-tumor or antitumor phenotype in animal models. Therefore, it has been proposed that inhibition of TGFβ signaling could result in TAN antitumor manifestation and that treatment with type I IFNs could induce antitumor polarization of TANs (Fridlender et al., 2009; Andzinski et al., 2016). Treatment with type I IFNs has been tested in various clinical trials (Ni and Lu, 2018). Also, TGFβ signaling inhibitors had been launched as an anticancer therapy in many clinical trials before the TGFβ immunomodulatory effects on neutrophils were revealed (Akhurst, 2017). The therapeutic approach currently tested in clinical trials includes targeting TGFβ signaling using specific antibodies or through molecules targeting its receptors (Morris et al., 2014; Herbertz et al., 2015). The human anti-TGFβ monoclonal antibody (GC1008) that neutralizes all isoforms of TGFβ was tested in a phase I clinical trial in patients with advanced malignant melanoma or renal cell carcinoma (NCT00356460), and the antibody showed antitumor efficacy with no dose-limiting toxicity at a dose of up to 15 mg/kg, with acceptable safety. The TGFβ antagonist, galunisertib (LY2157299), a small molecule inhibitor of the TGFβ receptor I kinase, was shown to re-sensitize drug-tolerant cells to anticancer therapeutics and demonstrated antitumor activity in animal models and was proposed as a strategy to improve the efficacy of immune checkpoint inhibitors (Huang et al., 2012). Galunisertib is being tested in clinical trials both as a monotherapy and in combination studies. In difficult-to-treat cancer types, such as glioblastoma (NCT01582269, NCT01682187, NCT01220271), pancreatic cancer (NCT0273416, NCT01373164), or hepatocellular carcinoma (NCT01246986, NCT02423343), combinations with chemotherapy, temozolomide-based chemoradiotherapy, and immunotherapy are being tested.

Different therapeutic strategies relying on the innate and adaptive immune systems are being investigated and hold great promise for oncology. In addition to the above described major clinical research achievements, other ongoing important clinical research will pave the way for new clinical trial approaches (Shaul and Fridlender, 2019). Improving our understanding of the role of neutrophils in the TME, neutrophil interaction with other immune components, and how tumor cells tune TANs toward their favor would have great impact in generating target-specific treatment strategies aimed at improving antitumor effects. Moreover, the emerging field of genetic engineering technology and *ex vivo* modification of immune cells could provide yet another avenue to improve the effectiveness of neutrophils in the fight against cancer.

## Combination Treatments Using Next-Generation Cell-Based Immunotherapies

Cellular therapy is a term representing several types of cell transplantation used clinically for patients with various types of cancer. Accordingly, the specific mechanisms of cellular therapy involved in the therapies are extensive. Antigen-specific immunotherapy is a therapeutic vaccine that directs tumor-specific immune cells to kill cancer cells (Schlom et al., 2007). Specific targeting for activation during therapeutic vaccination provides a powerful and low-toxicity benefit. However, cancer cells can evade the immune system through various strategies, such as dysregulation of T cell immune checkpoints, invasion, anti-apoptosis, outside environmental factors, and other nonspecific issues (Davies, 2014; Zhao and Subramanian, 2017). Synergistic combination therapies may provide the key to improving responses and reducing drug dosage and side effects for cancer patients. The combination therapies described below are listed in Table 1.

**TABLE 1:** Combination therapies using cellular therapy.

Cell type	Interventions/treatment	Targeted diseases	Phase	Trial no.
DC-based therapy	AML fusion DC + anti-PD-L1 (durvalumab)	AML	II	NCT03059485
	Autologous DC + anti-PD-L1 (avelumab)	Colorectal carcinoma	I/II	NCT03152565
	Autologous DC + anti-CTLA-4 (ipilimumab)	Melanoma	III/IV	NCT01973322
	Cryosurgery DC vaccination + anti-PD-1 (pembrolizumab)	Non-Hodgkin lymphoma	I/II	NCT03035331
	DC-CIK + anti-PD-1 (pembrolizumab)	NSCLC	I/II	NCT03360630
	TriMixDC-MEL + anti-CTLA-4 (ipilimumab)	Melanoma	III/IV	NCT01302496
	CMV mRNA-pulsed autologous DCs + anti-PD-1 (nivolumab)	Grade III/IV brain tumors	I	NCT02529072
	CMN-001 (CD40L RNA)-DC + anti-CTLA-4 (ipilimumab) + anti-PD-1 (nivolumab)	Renal cell carcinoma	II	NCT04203901
	Autologous DCs + TKI (dasatinib)	Melanoma	II	NCT01876212
NK-based therapy	NK cells + anti-HER2 (trastuzumab)	Breast cancer, gastric cancer	I/II	NCT02030561, NCT02843126, NCT02805829
	NK cells + anti-CD20 (rituximab)	B cell lymphoma	I/II	NCT02843061
	NK cells + anti-EGFR (cetuximab)	Head and neck cancer, NSCLC	I/II	NCT02507154, NCT02845856
	NK cells + anti-CD319 (elotuzumab)	Multiple myeloma	II	NCT03003728
	FATE-NK100 + anti-HER2 (trastuzumab) or anti-EGFR (cetuximab)	Advanced solid tumors	I	NCT03319459
	NK cells + anti-VEGF-A (bevacizumab)	Malignant solid tumors	I/II	NCT02857920
	NK cells + anti-GD2	Neuroblastoma	I/II	NCT03242603
	NK cells + anti-PD-1 (nivolumab)	Malignant solid tumors	I/II	NCT02843204
	NK cells + ALT803	Leukemia	I	NCT02890758
CAR-T cell–based therapy	CD30-CAR-T + anti-PD-1 (nivolumab, pembrolizumab)	Hodgkin lymphoma	I	NCT04134325
	CD19-CAR-T + anti-PD-1 (pembrolizumab)	Large B cell lymphoma	I/II	NCT02650999
	CD19 CD28-CAR-T + anti-CTLA-4 (ipilimumab)	B cell lymphoma, lymphocytic leukemia	I/II	NCT00586391
	JCAR014 + durvalumab	Non-Hodgkin lymphoma	I	NCT02706405

DC, dendritic cell; AML, acute myeloid leukemia; NSCLC, non-small cell lung cancer; TKI, tyrosine kinase inhibitor; NK cells, natural killer cells; EGFR, epidermal growth factor receptor; CAR, chimeric antigen receptor.

### DC-Based Combination Therapy

DC-based immunotherapy is the first cellular therapy to provide clinical benefit for patients with prostate cancer and has been tested in clinical trials in melanoma and hepatocellular carcinoma (Harzstark and Small, 2007). However, the clinical results were not as effective as expected. Most cancer types produce many factors that contribute to immune dysfunction, such as IL-6, IL-10, TGFβ, and VEGF, by inhibiting the function of DCs and T cells, and this resulted in poor clinical outcomes (Rabinovich et al., 2007; Melief, 2008). Therefore, the combination of DC therapies is a strategy to neutralize tumor-associated immune suppression and prolong the antitumor activity of DC-induced effector T cells. Blockade with anti-PD-1 monoclonal antibodies, anti-PD-L monoclonal antibodies, or anti-CTLA-4 monoclonal antibodies in combination with DC vaccination resulted in increased activation of cytotoxic CD8^+^ T cells and showed better treatment efficacy compared with monotherapy in mice (Sznol, 2012; Ge et al., 2013; Antonios et al., 2016; Coffelt and de Visser, 2016; Salmon et al., 2016). Recently, a selected number of phase I/II clinical trials have been initiated that combine DC vaccination in various malignant tumors with pulsing tumor-associated peptides and checkpoint inhibitors (anti-PD-1, anti-PD-L1; NCT03059485, NCT03152565). In a study of patients with stage III melanoma that progressed after DC therapy, administration of ipilimumab induced tumor-specific T cell responses (NCT01973322), which was not associated with an improvement in overall survival (Boudewijns et al., 2016). DCs pulsed with melanoma-associated antigens (MAGE-A3, MAGE-C2, and gp100: TriMix-DC) can induce expansion of antitumor T cells (Wilgenhof et al., 2016). Ipilimumab combined with the TriMix-DC vaccine therapy is under clinical follow-up and may be a more effective treatment for patients with advanced melanoma (NCT01302496). This indicates that combining DC therapy with CTLA-4 targeting agents could lead to synergistic effects. In mice that received DC therapy combined with tyrosine kinase inhibition, such as macrophage CSF receptor inhibitor, prolonged survival and improved CTL levels were observed compared with DC monotherapy (Dammeijer et al., 2017). DC therapy in combination with the tyrosine kinase inhibitor dasatinib has been shown to lead to increased recruitment of CD8^+^ T cell infiltration, and a clinical trial is currently ongoing (NCT01876212). A phase I study using recombinant human CD40L showed antitumor activity and long-term complete remission in patients with squamous cell carcinoma, and the current CD40L RNA-transfected DC combination therapy is in an ongoing phase II study for the treatment of renal cell carcinoma (NCT04203901) (Vonderheide et al., 2001). Various types of tumors are sensitive to DC vaccination and immune checkpoint blockade. Therefore, targeting both DCs and immune checkpoints can lead to promising strategies for next-generation vaccine combinations.

### NK Cell-Based Combination Therapy

As described above, NK cells are a type of cytotoxic lymphocyte and play a major role in host defenses against tumors and viral infections. NK cells are important in both innate and adaptive immune responses for potential cancer therapies (Terunuma et al., 2008). Adoptive cellular therapy using NK cells has been extensively studied in clinical trials, but its antitumor effect is limited. NK cell-based adoptive transfer has shown efficacy in the treatment of hematopoietic malignancies (Cheng et al., 2013). Combination therapy with antibodies and cytokines is required to obtain more potent tumor killing activity in transferred NK cells. Mainly TAA-targeting antibodies were used in clinical trials in combination with NK cell adoptive cellular therapy. Among the targeting therapeutic antibodies, rituximab targets CD20 in B cell lymphoma (Battella et al., 2016). Trastuzumab targets HER2 and is used routinely in combination with chemotherapy in HER2-overexpressing breast and gastric cancer (Maximiano et al., 2016). Trastuzumab is known to induce NK cell–mediated ADCC for tumor cell killing. In patients, the therapeutic efficacy of trastuzumab in the treatment of metastatic breast cancer has been demonstrated to induce ADCC by NK cells (Beano et al., 2008). Other targeting antibodies such as cetuximab, elotuzumab, and anti-GD2 are also associated with ADCC effects by NK cells and are currently under investigation in phase I or phase II studies in combination with NK cells (Taylor et al., 2009; Collins et al., 2013). NK100 is a first-in-class NK cell cancer immunotherapy consisting of adaptive memory NK cells, a highly functionally distinct NK subset. FATE-NK100 is undergoing clinical trials in combination therapy with anti-HER2 or anti-EGFR monoclonal antibodies against solid tumors (NCT03319459). A phase I/II study of the combination of anti-PD-1 monoclonal antibodies and NK cells for the treatment of malignant solid tumors is underway to determine the safety and efficacy of combination immunotherapy (NCT02843204). In patients with hematologic malignancies, ALT-803 (an IL-15 superagonist) combined with NK cells is being tested in an ongoing phase I trial. NK cell–based therapies have shown remarkable efficacy in some types of cancer, and combination therapies building on these results will likely prove beneficial for patients with cancer. In addition, NK cell adoptive cellular therapy combined with bispecific proteins is a new avenue of therapy being tested in ongoing clinical trials, the results of which will be of great interest to the field.

### CAR-T Cell Combination Therapy

CAR-T cells are genetically engineered to specifically recognize tumor cells, resulting in direct CAR-T activation and antitumor function (Eshhar et al., 1993). Most clinical studies have shown that CAR-T cell monotherapy had low clinical response in the treatment of solid tumors despite the progress made in treating hematologic malignancies (Kershaw et al., 2006; Lamers et al., 2016). Most solid tumors inhibit CAR-T activity through upregulation by immune checkpoint inhibitors such as PD-1. Therefore, using CAR-T cell therapy in combination with immune checkpoint inhibitors for patients with early solid tumors may demonstrate improved results compared with those seen with CAR-T cell monotherapy (Liu et al., 2016). In preclinical studies, CAR-T cell therapy with PD-1 blockade showed synergistic effects and improved long-term survival (John et al., 2013). In clinical trials, the combination of anti-PD-1 with CAR-T cell therapy enhanced the efficacy and persistence of CAR-T cells in the treatment of melanoma (Gargett et al., 2016). CTLA-4 can also be a good target to enhance CAR-T cell efficacy. Ongoing clinical trials examining the combination of CAR-T cells with PD-1 or CTLA-4 blockade are listed in Table 1. Although these trials are designed to treat solid tumors, combinations are applied to treat a variety of lymphomas, especially B cell lymphomas. In solid tumors, CAR-T cell therapy has low efficacy for T cell trafficking and infiltration into tumor lesions (Zhang et al., 2016). To address this problem, various methods are being studied, such as engineered CD6-based homing system CAR-T or combination therapy with tumor-infiltrating lymphocytes (Hu et al., 2018; Brown and Dustin, 2019). CAR-T cell-based combination therapies are possible and can improve the potential of CAR-T cell therapy. For this to be successful, it is important to determine which patients need a combination strategy and which combination is best for a given patient.

## Discussion

Checkpoint inhibitors and CAR-T cell therapy are currently the most prevalent immunotherapies for cancer patients. However, concerns exist about the relatively low clinical response to checkpoint inhibitors, the severe side effects of CAR-T cell therapy, and the limitations of cancer types for both therapies.

One major factor leading to resistance to checkpoint inhibitors is the so-called “cold tumor” microenvironment, mostly caused by lack of tumor-specific antigens, deficient antigen presentation, insufficient T cell activation, and a deficit of T cell homing to the tumor sites (Bonaventura et al., 2019). DCs, NK cells, B cells, and neutrophils are important cell components of the TME. DCs, as the professional antigen-presenting cells in the TME, once activated, can efficiently cross-present tumor antigens to T/B cells and thereby initiate T/B cell activation (Banchereau and Palucka, 2005). Mature DCs can also secrete cytokines to foster T cell response and release chemokines such as CXCL9, CXCL10, CCL3, CCL4, and CCL17 to recruit T cells into the tumor bed (Thaiss et al., 2011). Therefore, clinical agonists and antibodies targeting DC maturation, as well as various DC vaccines, can be the potential tools to transform a “cold tumor” to a “hot tumor” and synergize with checkpoint inhibitors for better clinical outcomes. Another advantage of DC vaccination lies in its lower systemic toxicity compared with other immunotherapies (Draube et al., 2011).

In addition to DCs, B cells in the TME can also present cognate TAAs to T cells (Sharonov et al., 2020). CD40 agonists, which activate both B cells and DCs, showed remarkable clinical benefits in melanoma patients when combined with checkpoint inhibitors (Bajor et al., 2018). Furthermore, B cells are the major cell type to produce antibodies to capture tumor antigens on the surface of tumor cells and to mediate ADCC by NK cells as well as antibody-dependent cell phagocytosis by macrophages (Tay et al., 2019).

NK cells, the first-line cytotoxic cells in the TME, can recognize and kill MHC class I negative tumor cells that can escape cytotoxic T cell–mediated destruction (Paul and Lal, 2017). CAR-T cells can also mediate MHC-unrestricted tumor cell killing (Benmebarek et al., 2019), but the clinical benefit is limited to patients with AML. Different from CAR-T cell therapy, CAR-NK cell therapy achieved preclinical and clinical efficacy in both hematologic and solid tumors (Burger et al., 2019). Another advantage of the CAR-NK strategy over CAR-T cell therapy is its low risk of inducing GVHD and cytokine release syndrome (Rezvani et al., 2017).

As for the pro-tumorigenic immune subsets, TANs promote the immune escape of tumor cells, contributing to the suppressive immune environment (Faget et al., 2017). To reverse the suppressive TME and reinvigorate cytotoxic T cells, therapies against TANs should be considered and may lead to better clinical outcome when combined with other immunotherapies. B cells, suppressive DCs, macrophages, and myeloid-derived suppressive cells also have roles in promoting tumor progression, which should also be taken into account (Awad et al., 2018; Largeot et al., 2019).

Challenges also exist in therapies targeting DCs, B cells, NK cells, and neutrophils. DC vaccination had limited clinical efficacy (Anguille et al., 2014). TLR/STING agonists lead to upregulation of the PD-1/PD-L1 axis (Blanco et al., 2008; Garcia-Diaz et al., 2017; Vatner and Janssen, 2019). STING signal is active not only in DCs and macrophages but also in T cells. Conversely, activation of STING in T cells leads to T cell stress and cell death (Larkin et al., 2017). The potential T cell defects caused by the STING agonist may reduce the clinical outcome of STING agonist–based immunotherapies. For CAR-NK therapy, CAR-NK cells must be irradiated to avoid a possible stimulation of GVHD, resulting in reduced cell life and antitumor efficiency (Kloess et al., 2019). The CD40 agonistic antibody may induce cytokine release syndrome and autoimmune reactions because CD40 is also expressed on platelets and endothelial cells (Vonderheide and Glennie, 2013). Treatment with the anti-Ly6G antibody is a common method for neutrophil depletion. However, anti-Ly-6G therapy also causes the depletion of monocytes and subsets of CD8^+^ T cells, limiting the clinical practice of this therapeutic strategy (Bao and Cao, 2011). All of these issues need further exploration for identification of possible solutions.

Various immune cell subsets exist and interact with each other in the TME. Therefore, one single immunotherapy may not be sufficient to reverse the immunosuppressive environment fostering tumor growth. Overcoming immune resistance may require an immunotherapy cocktail or combination of immunotherapy with routine cancer treatment (i.e., radiotherapy, chemotherapy, and surgery). Different cancer types have distinct immune signatures and TMEs (Thorsson et al., 2018). Immune signatures and TMEs also vary among individual patients. The optimal combination of various cancer therapies may depend on a thorough understanding of the individual’s immune signature. As such, personalized and combination immunotherapies may achieve unprecedented progress against cancer.

## Author Contributions

YS led the writing of the manuscript. KT designed the figures. CH supervised the study. All authors contributed to the writing and review of the manuscript.

## Funding

Scientific and financial support for the CIMAC-CIDC Network is provided through the National Cancer Institute (NCI) Cooperative Agreement U24CA224285 (to the MD Anderson Cancer Center CIMAC). This study was supported in part, by the Translational Molecular Pathology-Immunoprofiling lab (TMP-IL) at the Department Translational Molecular Pathology, the University of Texas MD Anderson Cancer Center.

## Conflict of Interest

The authors declare that the research was conducted in the absence of any commercial or financial relationships that could be construed as a potential conflict of interest.
